# Return of the pika: American pikas re‐occupy long‐extirpated, warm locations

**DOI:** 10.1002/ece3.9295

**Published:** 2022-09-14

**Authors:** Constance I. Millar, Andrew T. Smith

**Affiliations:** ^1^ PSW Research Station USDA Forest Service Albany California USA; ^2^ School of Life Sciences Arizona State University Tempe Arizona USA

**Keywords:** American pika, climate change, dispersal, *Ochotona princeps*, resilience

## Abstract

American pikas (*Ochotona princeps*), small mammals related to rabbits, occur in mountainous regions of western North America, where they live in shattered‐rock habitats (talus). Aspects of their physiology and life history create situations that appear to put pikas at risk from warming climates. Some low‐elevation, warm sites that historically harbored pikas have become extirpated, and the assumption is that these will not be re‐colonized under current climate trends. Unexpectedly, in 2021, we found that pikas had re‐colonized two very warm, low‐elevation, dry sites in eastern California, USA, in the Bodie Mountains and Mono Craters. Resident pikas appear to have been absent at both sites for ≥10 years. These findings suggest that pikas, which are normally diurnally active, are able to overcome thermal dispersal barriers and re‐colonize long‐extirpated sites, perhaps by moving during cool nights. Our data also highlight the often unrecognized suitability of pika habitat in warm regions where the interiors of taluses can remain stably cool even when external air temperatures are hot.

## Graphical abstract

Here we show two locations where American pikas were found in 2021 to have re‐colonized long extirpated, very marginal sites. The sites are in the Bodie Mountains and Mono Craters, eastern California, USA.

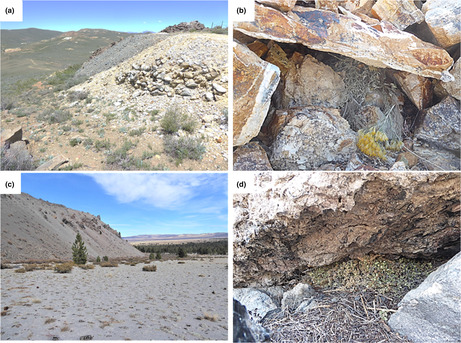

## 1 INTRODUCTION

**FIGURE 1:**
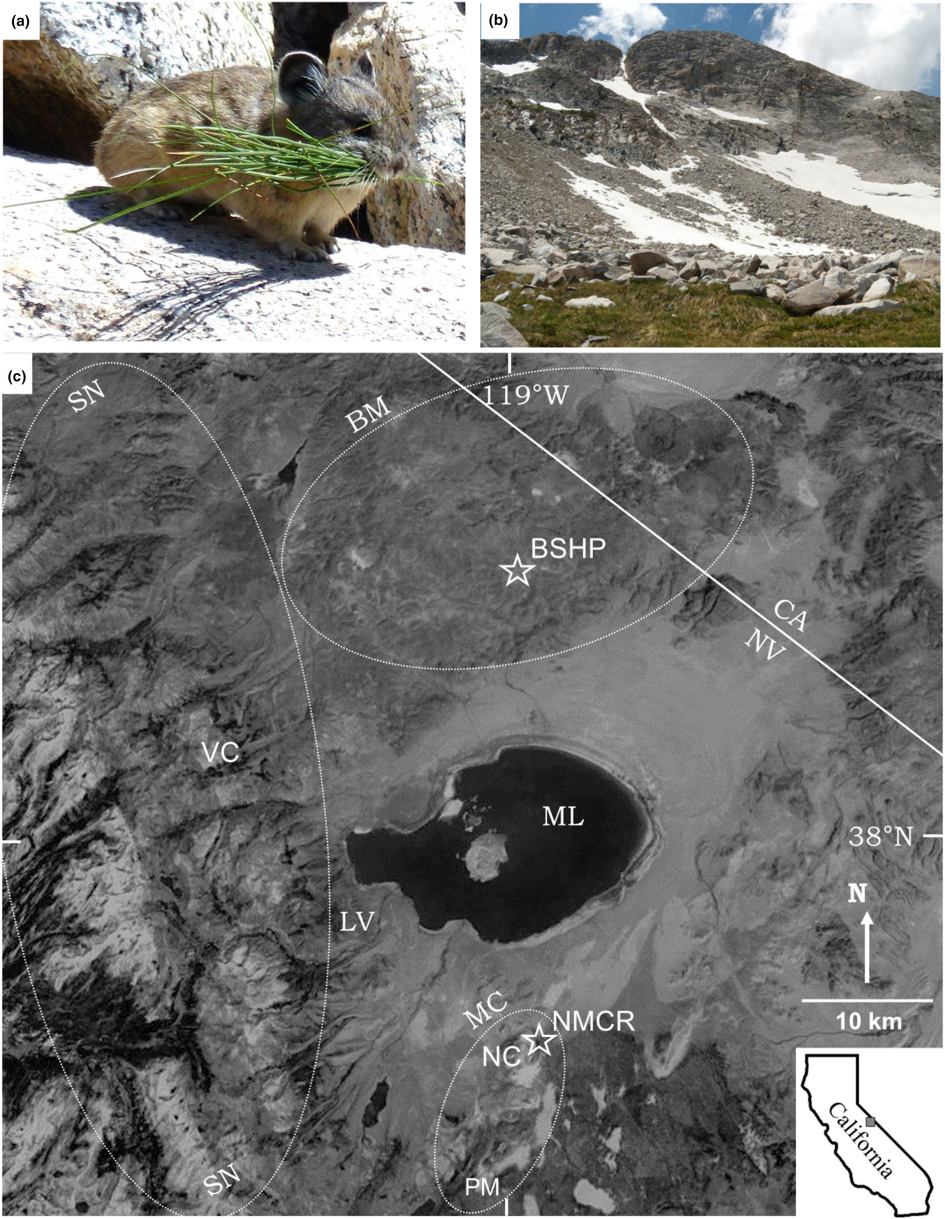
(a) American pika; (b) Typical talus habitat of alpine regions; (c) Map of the study area, Mono County, California; the two re‐colonization sites are identified by stars. Pika sites: BSHP, Bodie State Historic Park; NC, Northwest Coulee; NMCR, North Mono Craters; PM, Pumice Mine; VC, Virginia Canyon. Reference locations: BM, Bodie Mountains; LV, Lee Vining; MC, Mono Craters; ML, Mono Lake; SN, Sierra Nevada. Inset shows the location of the study region in eastern California.

The American pika (*Ochotona princeps*; Figure 1a) has gained notoriety as a bellwether of how warming climates may negatively influence species' populations (Beever et al., 2011; Ray et al., 2012; Smith, 2020; Smith et al., 2004), as reflected by both its historical and contemporary biogeography. The paleontological distribution of pikas across North America is one of contraction as climates warmed (Grayson, 2005; Smith, 2020). The lower elevational limit of modern‐day pika populations in the inter‐montane west is higher at warmer more southern latitudes and declines with increasing latitude where it is cooler (Grinnell, 1917). Because pikas have a poor physiological capacity to tolerate warm environments (MacArthur & Wang, 1973, 1974; Smith, 1974a), many fear that pikas will perish as air temperatures rise under contemporary climate change (Beever et al., 2003, 2011; Ray et al., 2012; Stewart et al., 2015; Wilkening et al., 2011). This concern derives from an assumption that pikas will be forced to move upslope as temperatures increase and eventually they will run out of space on mountain peaks, leading to population collapse and extirpation (McDonald & Brown, 1992; Ray et al., 2012).

Despite these widely repeated generalizations, important factors of pika ecology are routinely ignored that suggest a more nuanced and hopeful future for the species (Smith, 2020). Although pikas are commonly described as an alpine species, they are by no means high‐elevation obligates. While pikas occur abundantly on high mountains, often above local tree line and extending to peaks as high as 4300 m (Figure 1b), what is often overlooked is that they may occupy their obligate habitat, talus or piles of broken rock (Smith & Weston, 1990), far downslope. Where suitable rocky environments occur, pikas can be found at mid‐montane elevations and lower, such as in semi‐arid sagebrush (*Artemisia* spp.) steppe communities as low as 1450 m in the Great Basin (Jeffress et al., 2017; Millar et al., 2013, 2018; Smith et al., 2016). Pikas even occupy talus near sea level in the Columbia River Gorge, Oregon (Horsefall, 1925; Simpson, 2009).

Conventional measures of ambient temperature fail to capture the temperatures encountered by pikas during their daily activity (Millar et al., 2016), although they are routinely used as proxies. The rocky habitats of pikas have surprising and little known thermal properties. Unique ventilation processes keep talus interiors stably cool in summer and warm in winter relative to outside air temperatures (Millar, Westfall, et al., 2014; Morard et al., 2010). Behaviorally, pikas adjust their activities in response to outside air temperatures. Although primarily considered a diurnally active species, pikas avoid high summer daytime heat by taking refuge within the cool talus, becoming crepuscular (Otto et al., 2015; Smith, 1974a; Smith et al., 2016). Pikas may even forage actively at night (Camp et al., 2020; Hall & Chalfoun, 2019; Millar & Hickman, 2021; Smith, 1974a; Smith et al., 2016).

Within each patch of talus, pikas are individually territorial, and male and female territories tend to alternate spatially (Smith & Ivins, 1983, 1984). Their territories are large (400–700 m^2^; Smith & Weston, 1990), resulting in a low density of pikas at any locality. As with all species, there is a probability that pikas, particularly those with small populations on an isolated patch, will become extirpated – whether caused by increasing temperatures, predation, or stochastic processes (Harrison, 1991; Lande, 1988; Smith, 1974b). Many have proposed that increasing temperatures caused by global warming may impede the re‐colonization of formerly pika‐occupied patches (reviewed in Smith, 2020). This is particularly likely in pikas, as they are notoriously poor dispersers (Peacock & Smith, 1997; Smith, 1987; Smith & Ivins, 1983). The extreme heat that characterizes dispersal corridors between suitable pika habitats in warm localities leads to a low probability of pika re‐colonization (Millar et al., 2016; Smith, 1974a, 1974b). In his comprehensive review of the conservation status of pikas, Smith (2020) concluded: “The trait that puts pikas most at risk from climate change is their poor dispersal capacity.”

Further challenges to understanding pikas' response to changing climates relate to the difficulty in accurately estimating site occupancy versus extirpation. Low animal densities, below‐ground habitats, and metapopulation conditions can lead to errors, usually under‐estimates, of site occupancy, especially from rapid surveys (reviewed in Smith, 2020). In some Great Basin locations, pikas have been found to scatter‐hoard (small amounts of cached food distributed throughout territories) rather than to forage at central places (large haypiles in the center of territories), leading to difficulties in the interpretation of population conditions (Smith et al., 2016). Stochastic loss of animals and subsequent re‐colonization of small, disjunct habitat patches from large “mainland” population sources means that there will be years when small patches might be empty and misevaluated as metapopulation extirpation (Smith & Nagy, 2015). Thus, the most secure estimates of pika population persistence or extirpation require repeat surveys over time.

This note describes two surprising and unexpected re‐colonization events by pikas at low elevation, exceptionally warm environments in eastern California that call into question previous conclusions about pika dispersal. We refer to climatic margins herein as extreme values that have been previously measured using proxy indicators (i.e., standard air temperature) for extant vs. occupied pika sites (Millar et al., 2018).

## 2 RE‐COLONIZATION OF FORMERLY EXTIRPATED PIKA SITES

### 2.1 Bodie State Historic Park

#### 2.1.1 Background

**FIGURE 2:**
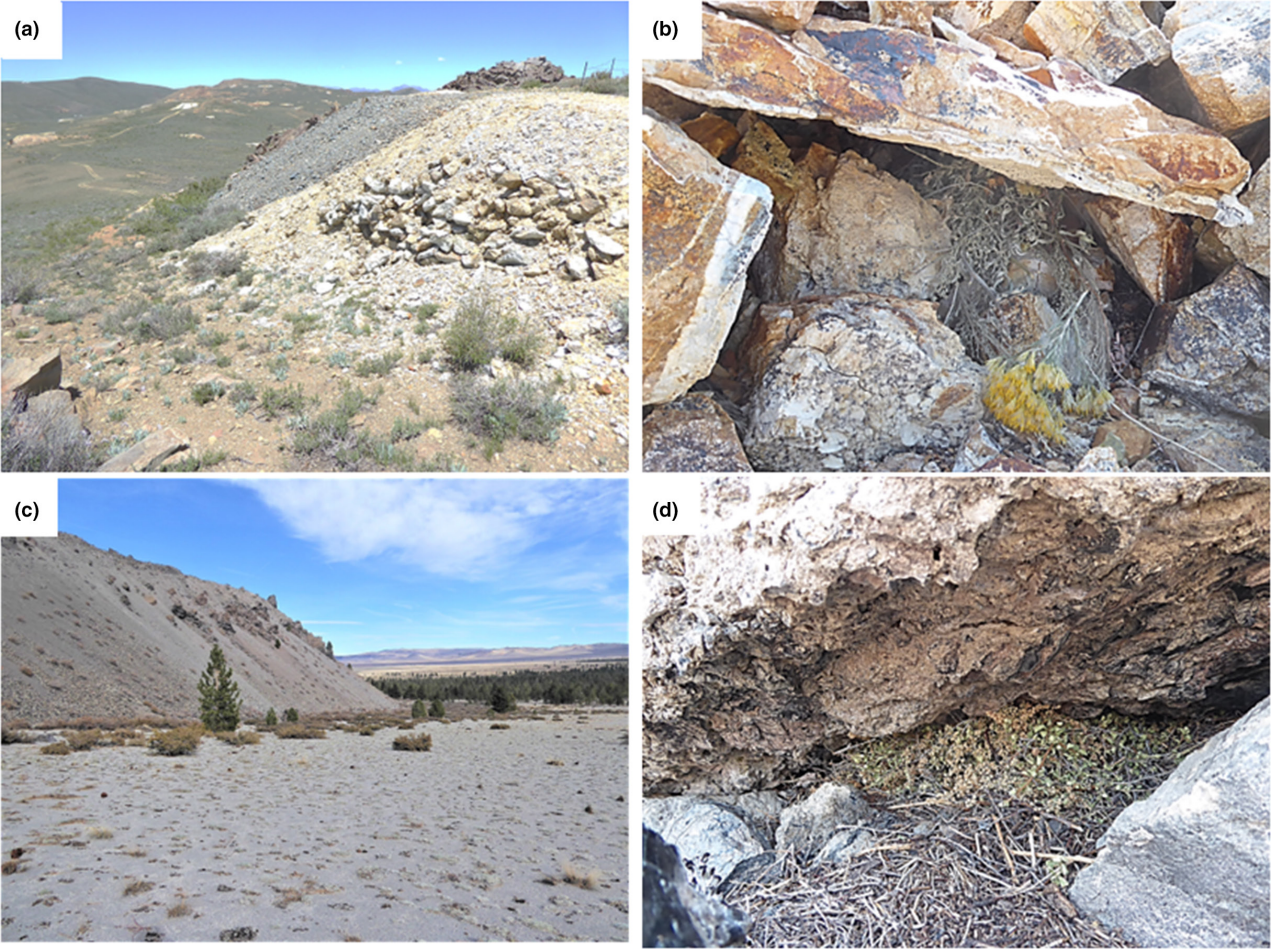
Study sites, Mono County, California. Bodie mountains: (a) Bodie State Historic Park; ore‐dump pika habitat, view north across Noonday Mine, a southern constellation of sites looking toward northern constellation; (b) Fresh haypile at Noonday Mine occupied in 2021. Mono Craters: (c) View of lava flow habitat where pikas have re‐colonized; (d) Fresh haypile at North Mono Craters.

The Bodie Mountains are a small mountain range in the western Great Basin east of the Sierra Nevada (Figure 1c). Relief is low and the mean elevation of the upland plateau lies between 2400 and 2800 m. Native pika habitat is poor in quality and scattered across the range, but evidence of the previous occupation by pikas, now long extirpated, is distributed widely (Millar, Heckman, et al., 2014). At present, only two sites are known to be occupied by pikas, one in the native habitat at the north end of the range (Millar et al., 2013) and another 20 km south in anthropogenic habitat. At the latter site, Bodie State Historic Park (BSHP; 2530–2695 m), Smith has been studying pikas in the ore dumps of the mining region since 1969 (Smith, 1974a, 1974b, 1978, 1980; Smith & Gilpin, 1997; Smith & Nagy, 2015; see Figure 2a). Vegetation at BSHP consists of a semi‐arid sagebrush steppe community.

The first full census of the Bodie metapopulation was completed in 1972 and consisted of 76 isolated ore dumps and 3 “mainland” sites (relatively continuous habitat partially sampled). Censuses were conducted in late summer when the pika population was at its maximum following summer reproduction. Occupancy of territories on each ore dump patch was determined by sign: fresh distinctive small round fecal pellets deposited by pikas and/or the presence of a fresh green haypile. Pikas do not hibernate and accumulate caches of vegetation (haypiles) during summer to serve as a source of food over winter (Conner, 1983; Dearing, 1997; Smith & Ivins, 1984). The study area is mildly constricted in the middle, demarcating a “northern” and a “southern” constellation of patches. This distinction is made purely for convenience – the patches in the Bodie pika metapopulation are scattered across 3 km north to south, and only 150 m separate the southernmost patch in the north from the northernmost patch in the south. The number of ore dump patches is similar in the north versus the south, but all three “mainlands” and seven of the nine largest patches are in the north. The two large patches in the south are the Noonday and Red Cloud mines (see Smith, 1974b; Smith & Gilpin, 1997; Smith & Nagy, 2015). Additional censuses of all ore dumps were conducted in 1977, 1989, 1991, 1992, 1993, 1994, 1995, 1996, 1997, 1998, 1999, 2000, 2001, 2003, 2004, 2005, 2006, 2008, 2009, and 2010 (Smith & Nagy, 2015; White & Smith, 2018).

The repeated censuses of occupancy on the patchily distributed ore dumps portrayed the Bodie pikas as an excellent example of a classic metapopulation system (Smith & Nagy, 2015; White & Smith, 2018). Populations on patches frequently went extinct (inversely related to patch size) and re‐colonizations occurred (inversely related to inter‐patch distance). Between 1989 and 2010, there was an almost equivalent number of extirpations (114) and re‐colonization events (109). However, an early trend was a general decline in the occupancy of patches in the southern constellation (Moilanen et al., 1998; Smith & Gilpin, 1997). In 2006, only three pikas at the Noonday Mine and one pika at the northernmost ore dump were found in the southern constellation, and no southern patches were occupied in annual censuses through 2010 (Smith & Nagy, 2015). Nichols et al. (2016, p. 164) presents a map, based on annual censuses of all ore dump patches at Bodie conducted from 2010, claiming that the 37 patches comprising the southern constellation were continuously unoccupied from 2006 until the time of their publication (but see below). Based on Nichols et al. (2016) report Stewart et al. (2015) claimed the southern constellation at Bodie as one of their historical 10 documented pika population extirpations.

#### 2.1.2 Temperature and climate

Bodie State Historic Park is a relatively hot climate for pikas (Smith, 1974a; Smith & Nagy, 2015). Complete daily maximum temperatures are available for the summer months (June, July, and August) for 84 years between 1895 and 2010. July was the hottest month, with an average monthly maximum temperature of 24.8°C. During this time period, the average monthly maximum temperature in summer months increased by approximately 1°C, and there was extreme variability among years in average monthly maximum temperatures as well as the number of extremely hot days (≥25 or ≥28°C) within a summer (Smith & Nagy, 2015).

Although pikas are responsive to temperature relations in their environment (Otto et al., 2015; Smith, 1974a), Smith and Nagy (2015) found no relationship between the number of extirpations of pikas on ore dump patches or of re‐colonization events with temperatures encountered during the current or previous year. Extirpations were not more common in hot years (measured by either monthly average maximum temperatures or by the number of extreme daily temperatures exceeding 25 or 28°C), and re‐colonization events were not more common when these temperature metrics were low or infrequent (Smith & Nagy, 2015).

#### 2.1.3 2021 Census

In early October 2021, we repeated the standard survey of all BSHP ore dump patches, marking the 50th anniversary of the first census and 11 years since the last complete census conducted by Smith (Smith & Nagy, 2015). We observed, as expected, widespread occupation of the northern mainland patches. To our surprise, we found three active territories in one of the more distant patches of the southern constellation (Noonday Mine), 1.4 km from the nearest occupied patch in the northern constellation. The occupation was determined by seeing a pika, hearing diagnostic calls, the occurrence of fresh fecal pellets, and presence of widely separated fresh (green) haypiles tucked into the ore dump (Figure 2b).

Other observations of pikas in the southern constellation were made prior to our 2021 observations. Nichols et al. (2016, p. 167–168) mention seeing a pika in 2015 at an undisclosed site 1.2 km from “the nearest occupied patch” – most likely the Red Cloud Mine, 400 m from the Noonday Mine (thus, the contention earlier in Nichols et al., 2016 that the southern constellation was extirpated at that time was incorrect). Klingler (2017) further elaborated that in 2016, she found five pikas at the Red Cloud and one additional pika in a smaller ore dump, the Booker Consolidated shaft, 900 m further south.

Our observations in 2021 of pikas on the Noonday Mine, coupled with those of pika occupancy on the Red Cloud Mine and the Booker Consolidated shaft (Klingler, 2017; Nichols et al., 2016), indicate that following nearly a decade of extirpated status, there have been multiple re‐colonization episodes from the northern constellation to the southern constellation.

### 2.2 Mono Craters

#### 2.2.1 Background

The Mono Craters, a small range of young volcanic cones south of Mono Lake and ~35 km distant from BSHP (Figure 1c), has a different kind of extreme habitat for pikas than at BSHP. The barren volcanic domes and coulees are steep, have unstable slopes that terminate abruptly at the edge of the flows, and are largely devoid of vegetation. Tephra that erupted from the vents covers adjacent ground surrounding the Craters, in which scattered Jeffrey pines (*Pinus jeffreyi*) are interspersed with sparse, dry, shrub vegetation. Many tephra talus slopes occur from low to high elevations within this range.

A. T. Smith (unpublished observations) first encountered pikas in the Mono Craters in 1969, and subsequently located an active population in the 1990s at the Pumice Mine site (Figure 1c; 2565 m) in the southern reaches of the Craters. We have informally monitored this site since that time. Between 1969 and 2008, we visited the site to determine the presence of pikas (animals seen or heard) every 1–3 years; since 2008, we have evaluated occupancy annually in the same manner. A detailed behavioral ecology study was conducted at the Pumice Mine site on a reach of tephra with nine pika territories in 2012 and 2013 (Smith et al., 2016). We have documented a persistent population at the Pumice Mine site through 2022, and occupied pika territories extend both west and east of our study area.

C. I. Millar (unpublished surveys) has extensively explored talus habitat throughout the Mono Craters since 2009 using widely accepted methods for evaluating occupancy as follows: current occupancy of a talus (pika seen or heard and/or presence of current‐year haypile vegetation), former occupancy (only old haypile vegetation or dried, decomposing fecal pellets), or no indication of occupancy (none of these signs; Beever et al., 2003; Jeffress et al., 2017; Millar et al., 2018; Simpson, 2009; Wilkening et al., 2011). Although repeat surveys were not made at all sites in all years, these opportunistic observations revealed old pika sites to be widely distributed throughout the talus habitat of the range. In 2011, a single pika‐occupied site, as determined by a fresh green haypile, was found at an internal talus edge (NW Coulee; 2301 m) near the north end of the Craters (Figure 1c). This site has not shown signs of occupancy since 2016.

At the far north end of the Mono Craters, 7 km from the Pumice Mine site, Millar (Millar & Hickman, 2021; Millar et al., 2013; C. I. Millar, unpublished observations) has been monitoring the presence of pika sites since 2011 (North Mono Craters, NMCR; 2092–2260 m; Figures 1c and 2c). In this area, opportunistic sampling surveys were repeated every 1–2 years during the haying season (mid‐summer to early fall) using the methods described above. While evidence of prior occupancy (decomposing haypiles and sparse old fecal pellets) was found in sites along this margin, over the survey years the only recent evidence of a pika in this region was from three camera‐trap photos of one animal in August 2018, where the camera faced an old haypile (2216 m; Millar & Hickman, 2021). The animal appeared briefly, running through the scene; we were unable to determine whether or not the animal was a resident, but no signs of such were present.

#### 2.2.2 Temperature and climate

Like Bodie, the Mono Craters represents a very hot environment to support populations of pikas, particularly in comparison with temperatures found in the nearby Sierra Nevada that include “typical” pika sites in subalpine and alpine environments. The average monthly maximum temperature in July at the Pumice Mine site was 22.8°C, and at the NMCR site was 25.4°C (Millar et al., 2013; see also Millar & Hickman, 2021; Smith et al., 2016).

**FIGURE 3:**
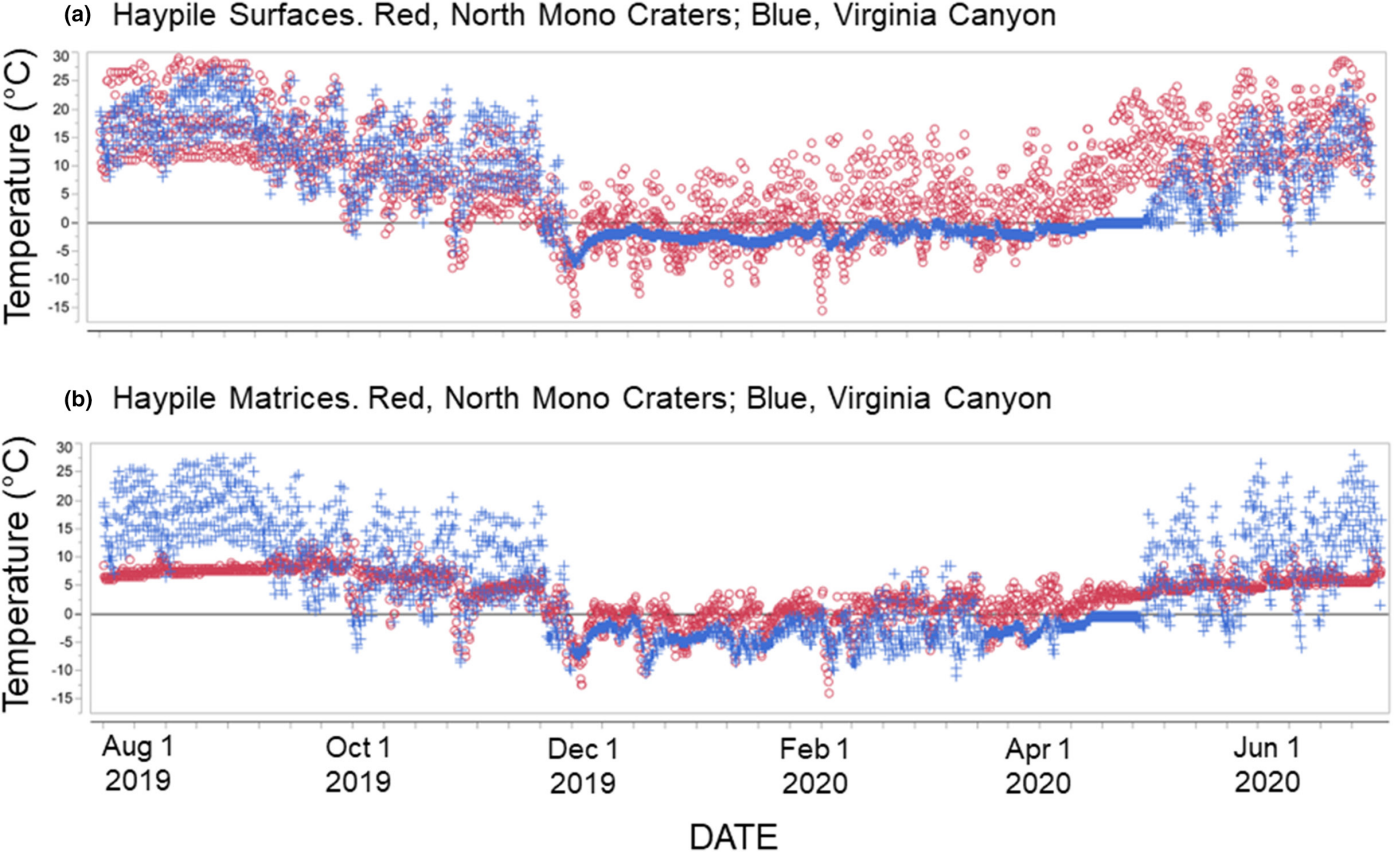
Temperatures measured by dataloggers at active pika sites, summer 2019 to summer 2020. North Mono Craters (2219 m; red points) and Virginia Canyon, Sierra Nevada (3182 m; 25 July 2019–12 July 2020; blue points). Maxim iButtons model 1921G, measured at 4 h intervals starting at noon. (a) Temperatures at surface of talus/lava at haypile site; (b) Temperatures within matrix of talus/lava (0.5–0.75 m below surface).

Whereas the only temperature data we have at Bodie come from a standard weather station, in the Mono Craters we have been monitoring contextual temperatures for many years (along with sites widely spaced along the eastern Sierra including other warm and lower elevation sites) using mini‐dataloggers (Maxim iButtons thermochrons; Millar & Westfall, 2010; Millar, Westfall, et al., 2014; Millar et al., 2016). Temperature curves from thermochrons positioned on a NMCR haypile surface from mid‐summer 2019 to mid‐summer 2020 (Figure 3a, red) show warm summer days and a snow‐free winter (as demonstrated by the high variability in daily temperatures without a blanket of snow to buffer the temperature extremes). Considering the warmest part of summer days (1200 and 1600 h), mean temperature at the NMRC haypile surface (33.2°C) was far higher than considered tolerable for direct exposure by pikas, with extremes even greater. By contrast, temperatures within the lava interior (matrix, 0.5 m below the haypile) were cool (mean 7.3°C), and highly stable throughout the course of the summer day and throughout the warm season (SD 1.5°C; Table 1).

**TABLE 1:** Summary of mean temperatures and standard deviations (SD) during summer (15 June–15 September; measurement period 2018‐2020) from data loggers at the North Mono Craters site (re‐occupied in 2021) and five mid‐elevation sites (continuously occupied “typical” pika sites in the adjacent Sierra Nevada).

Pika sites	Elevation (m)	Summer temperatures °C							
		Mid‐day		SD		Night		SD	
		Surf	Mx	Surf	Mx	Surf	Mx	Surf	Mx
North Mono Craters (1 site)	2195	33.2	7.3	4.1	1.5	12.4	6.7	4.2	1.0
Sierra Nevada (5 sites)	*X* = 2835	24.4	16.9	6.3	4.3	15.4	15.9	3.9	4.1

*Note*: Temperatures are given for the talus/lava surface (Surf) at a resident pika's haypile and the talus/lava matrix (Mx; 0.5–0.75 m below the talus surface). Summer mid‐day readings taken at 1200, 1600 h; Summer night readings taken at 2000, 2400, 0400 h.

Comparing temperatures at NMCR to the same years at pika haypiles in five typical Sierra Nevada mid‐montane sites (mean elevation = 2835 m; Millar & Hickman, 2021) shows important differences in habitat conditions between the environments (Table 1). Differences between NMCR and the Sierran sites were especially pronounced for summer midday and night temperatures, where mean midday surface temperatures were nearly 10°C warmer at NMCR than at the Sierran sites (33.2 vs. 24.4°C). By contrast, the mean matrix temperature for midday at NMCR was nearly 10°C cooler (7.3 vs. 16.9°C), a pattern that persisted for summer night temperatures as well. Again, matrix temperature stability during summer was much greater (lower SD) at NMCR than for the Sierran sites (Table 1).

Differences between the Mono Craters and Sierran sites can be visualized by temperature curves plotted for NMCR and a typical mid‐elevation Sierran pika site (Virginia Canyon; 3182 m), 30 km distant from NMCR (Figures 1c and 3, blue). These show higher temperatures for the surfaces at NMCR (Figure 3a), including through the winter and the lack of snow at NMCR that year, as well as the remarkably stable, low matrix temperature (Figure 3b).

#### 2.2.3 2021 Observations

In summer 2021, while conducting routine surveys of presumed unoccupied habitat in the Mono Craters, Millar observed an unexpected re‐colonization by pikas at NMCR, the previously assumed extirpated, low‐elevation site. This finding included abundant signs of active pika occupation: observing pikas, hearing their characteristic vocalizations, and finding fresh green haypiles (Figure 2d). These constituted an estimated 10 territories that extended across 1.3 km of lava‐flow edge from 2145 to 2250 m, and included the haypile where the camera trap was installed in 2018 (Millar & Hickman, 2021). Vegetation in the haypiles included sparsely distributed plant species growing adjacent to the lava flow, and consisted mostly of shrub (*Ribes cereum*, *Ericameria nauseosa*, and *Holodiscus discolor*) and conifer foliage (*P. jeffreyi*), with almost no herb species. This NMCR site represents the lowest elevation of an occupied pika site we know of in Mono and Inyo Counties, California.

## 3 POSSIBLE CAUSES FOR UNEXPECTED RE‐COLONIZATIONS BY PIKAS

The narrative that American pikas are on a trajectory toward extinction has largely been proposed based on investigations of a small number of historically known pika populations (Great Basin – 25 sites: Beever et al., 2003, 2011; Wilkening et al., 2011; California – 67 sites: Stewart et al., 2015). Analyses of climatic variables at sites formerly occupied by pikas were compared with extant sites, and it was determined that high summer temperature was the primary driver of extirpations. However, a more comprehensive dataset from 2387 pika sites in the Great Basin found that sites previously occupied by pikas occurred within the same climate space as extant sites (Millar et al., 2018).

The key element in these analyses is the portrayal of sites historically known to contain pikas as being currently unoccupied. While such sites could represent a local patch extirpation, there is also the possibility of false negatives – the failure to detect pikas at these sites. Numerous examples of false negatives occur in the pika literature (reviewed in Smith, 2020). For example, a patch classified as extirpated in Beever et al. (2003) was later reported to contain a robust pika population (Beever et al., 2011). Failure to detect pikas is increased in marginal (low‐elevation, hot) habitats (Smith, 2020). The primary sign used to determine pika occupancy is siting or vocalization of an animal and/or the presence of fresh characteristic fecal pellets and a fresh green haypile. However, at the Pumice Mine site, neither pellet piles nor haypiles were found across the width of the behavioral ecology study area inhabited by nine pikas; only one characteristic haypile was present earlier at the site in 2011 (Smith et al., 2016). Pumice Mine pikas were also infrequently active (seen in only 10.3% of all minutes of observation); the site could be observed for hours without seeing any of the pikas known to be present (Smith et al., 2016). Even repeated surveys could falsely characterize this site as vacant. Similar situations occurred in the comprehensive study of pika occupation across the Great Basin, where sites at climatic margins, especially very warm and low‐elevation areas, showed little indication of central‐place foraging and more scatter hoarding, which is more difficult to detect than classic large, central haypiles. Pikas at these sites also were less surface active during midday (Millar et al., 2018).

An additional possibility is that pikas can re‐colonize historic sites that were determined to be extirpated. Our observations in 2021 found two such re‐colonization events. Multiple experienced pika biologists censused the southern constellation of patches at Bodie, including the Noonday and Red Cloud mines, and failed to find any current pika occupation from 2006 to 2015. Since 2015, however, it appears there have been several colonizations of this region. The results of Stewart et al. (2015), which include the Bodie southern constellation as an example of a pika site extirpation, have even been published in one of the most popular university majors’ biology textbooks (Urry et al., 2020) – but our observations show that the site is currently occupied.

Repeat surveys at the NMCR site in the Mono Craters found no evidence of occupation until the summer of 2021 (Millar et al., 2013; Millar & Hickman, 2021; C. I. Millar, unpublished observations). Extension of these surveys documented that habitat along the perimeter of the Craters is abundant and that there is evidence of many old (apparently unoccupied) pika territories. Although we know of only one other occupied site at the south end of the Craters, the potential exists for other active pika sites near NMCR that could have been the source for the re‐colonization observed in 2021 – such as the apparently transient individual observed via our camera trap in 2018 (Millar & Hickman, 2021). The large number of territories appearing there so quickly remains difficult to explain. Even if each haypile does not represent one animal, there was still an indication of multiple animals re‐occupying this site, apparently over ~1–2 years. Since pikas do not disperse long distances, we have to assume there is a nearby undetected extant population that served as a source for dispersing juveniles to NMCR. Our 2018 camera‐trap transient gives support to such speculation.

Our survey results during the summer of 2021 show that caution should be taken when labeling a site extirpation. Vigilance is particularly important when censusing low‐elevation, hot sites. It is also important to evaluate climate limits for pikas relative to what they encounter as small mammals, rather than comparing standard meteorological values (Millar et al., 2016; Millar & Westfall, 2010). By conventional air temperature standards, taken at ~2.5 m height above the ground, the BSHP and Mono Craters sites lie outside the range many authors have estimated as necessary for pika persistence (e.g., Beever et al., 2003; Hafner, 1993; Simpson, 2009; Wilkening et al., 2011) – by these measures, they are truly marginal sites. But, considering the temperature conditions that pikas actually encounter in their habitat, their persistence and ability to disperse (to re‐colonize vacant habitat) suggest a different outcome (Millar et al., 2016). Although the lava surfaces where pikas live get extremely hot on summer middays, the lava matrices stay remarkably and stably cool. Relative to other talus substrates, lava habitat has been shown to have exceptionally cool and unvarying temperatures, including at the South Mono Craters, Inyo Craters, and elsewhere (Smith et al., 2016). At the Mono Craters, large bodies of persistent and deep‐lying ice have been documented during geology explorations and mining operations; subfreezing temperatures persist below the hot surfaces (Loney, 1968). Unusual ventilation processes in talus and related landforms, which lead to partial decoupling of internal air temperatures and their variances from outside air, appear to maintain these conditions (Millar, Westfall, et al., 2014; Morard et al., 2010). These thermal characteristics of lava, combined with the behavioral capacity of pikas to be selectively active day or night, suggest that the Bodie ore dumps and Mono Craters talus remain suitable habitats for pikas, even as external air temperature rises. For example, minimum (night) air temperatures in the eastern Sierra Nevada region have risen nearly 2°C over the past 120 years (figure 6e in Millar et al., 2020), yet pikas persist in many of the warmest sites, in part related to cool talus interiors. Concerns for pika declines are often based on external air temperature trends such as this, without taking into consideration the stable “refrigeration” nature of taluses.

The additional requirement for the observed re‐colonizations, that is, of pikas dispersing across what appears to be very hot environments where no ameliorative thermal processes exist (Millar et al., 2016), also comes as a surprise. Both of us have issued concerns that even if talus habitats remain cool enough temperatures for pika persistence, the increasing heat of dispersal areas between taluses may well be the key factor for population extirpations in the future, especially at warm and low‐elevation sites. The finding to the contrary here suggests that pikas are able to circumvent this challenge, possibly by constraining their movements to nights and cool days.

The findings reported here of unexpected re‐colonization, especially at warm, low‐elevation sites near the apparent climatic limits of pika habitat, raise important questions of how extirpation and occupancy – and at what spatial scale – should best be evaluated. For species such as pikas, where inter‐annual site turnover is documented to be high, and where previously assumed extirpated sites are found to have been falsely evaluated as extirpated, perhaps a new term needs to be introduced for situations where “true” extirpation has not (yet) been determined. This might be an interim assignment of status until a more confident decision after some agreed upon number of years becomes warranted. From our study, we suggest that a ~10 year period might be too short an interval for reporting extirpation; rather during such a period, researchers might designate an apparently unoccupied site as an “extended absence,” which would highlight the need for re‐surveys. This issue is also relevant to modern re‐surveys of historic reports (e.g., from the early 20th century), where multiple re‐surveys over ~10 year period would be prudent. We offer this as an important question for future research.

## AUTHOR CONTRIBUTIONS

**Constance I. Millar:** Conceptualization (equal); data curation (equal); formal analysis (equal); investigation (equal); methodology (equal); project administration (lead); writing – original draft (lead); writing – review and editing (equal). **Andrew T. Smith:** Conceptualization (equal); data curation (equal); formal analysis (equal); investigation (equal); methodology (equal); project administration (supporting); writing – original draft (supporting); writing – review and editing (equal).

## CONFLICT OF INTEREST

None declared.

## Data Availability

Data are archived at Dryad: https://doi.org/10.5061/dryad.rr4xgxdc0.
